# A high-level, simulator independent, Python library for simulating small networks of multicompartmental neurons

**DOI:** 10.1186/1471-2202-13-S1-P13

**Published:** 2012-07-16

**Authors:** Michael Hull, David Willshaw

**Affiliations:** 1 Institute for Adaptive and Neural Computation, University of Edinburgh, EH8 9AB, UK

## 

We present a high level, simulator independent, Python library for building simulations of small populations of multi-compartmental neurons, in which membrane voltage is calculated from individual ionic current flows.

The API allows the user to quickly construct simulations of small populations of neurons and synaptic connections, with particular focus on: (1) allowing simulation specification, with visualisation and analysis in a minimal, clean, human readable language; (2) reduction of complex simulation tool chains to a single python script; (3) promoting reproducible research through automatic self-documentation; (4) encourage the re-use of morphologies, neurons and channels in a way that specific and stochastic variation in parameters is simple; (5) transparent handling of different units; (6) allowing the use of established formats, (e.g. NEURON nmodl files[1]); (7) simplifying the definition and sharing of new channel types, including the possibility to support other libraries and standards easily (e.g. PyNN[2], 9ML, NeuroML[3]).

Consider the following example: “We would like to build a network, of 30 neurons. Each soma should be 20(± 3) µm diameter, and axons are 1000µm long, have a hillock of radius 5um for the first 10µm, then taper to a radius 0.3µm. We will use a single compartment for the soma, divide the hillock into compartments of length 1um, and the axon into 100 compartments. We will use the sodium and potassium voltage-gated channels as specified by the equations and parameters in paper X, but use 3 times the density of sodium channels on the hillock. The neurons should be connected by gap junctions, distributed according to algorithm Y, with resistances of 1±0.5G Ohm. The neurons also excite other neurons in the population with AMPA synapses, with a probability of connections between any pair of 20%. We will stimulate this network with NMDA synaptic input to ~50% of the neurons at fixed times, as well as different levels of step current injections to cell 12, and plot graphs of the ionic currents flowing across the membrane of cells 2, 12, and any cells that spike, as well as the membrane voltage of all the neurons. We want to compare with the case when the gap junctions have been blocked.”

The library allows the specification of this complete simulation and visualisation of the results in a single Python file of less than 100 lines, only requiring a single command to run. It is designed to make it possible to specify connection algorithms, morphologies, channel descriptions and simulation parameters as high-level objects, such that they can be reused in other simulations, without the need to copy and paste code. Units are handled transparently, reports of the simulation setup are automatically generated, and a clean separation between of simulation and visualisation is achieved through a mechanism of 'tagging' in the framework. The library is based on standard Python libraries, (numpy, matplotlib), making it easy to integrate with other libraries.

The framework is under active development, with a NEURON backend, currently supporting everything in the above example. A first release is intended for July 2012.
